# Resilience and self-esteem in Tunisian women: A study on psychological strength

**DOI:** 10.1192/j.eurpsy.2025.1613

**Published:** 2025-08-26

**Authors:** H. Mhiri, I. Chaari, I. Mannoubi, N. Boussaid, F. Charfeddine, L. Aribi, N. Messedi, J. Aloulou

**Affiliations:** 1 Psychiatric department “B”, Hedi Chaker University Hospital, Sfax, Tunisia

## Abstract

**Introduction:**

Self-esteem, defined as an individual’s overall sense of self-worth and confidence, plays a crucial role in shaping how people interact with their environment. It has been strongly linked in the literature to both mental health and resilience. Tunisian women, known for their achievements despite numerous challenges, provide a compelling population for exploring the dynamics between self-esteem and resilience.

**Objectives:**

To assess the impact of self-esteem on resilience among Tunisian women.

**Methods:**

This cross-sectional study surveyed Tunisian women aged 18 and above, between June and August 2024, using an online questionnaire. General health information was gathered through a structured survey. Self-esteem levels were evaluated using the Rosenberg Self-Esteem Scale, while resilience was measured with the 25-item Connor-Davidson Resilience Scale (CD-RISC 25).

**Results:**

Data were collected from 695 Tunisian women, with participants having a mean age of 36.72 ± 12.23 years. Of the respondents, 22.3% reported using psychoactive substances: 20.4% smoked, 9.4% consumed alcohol, and 2.5% used cannabis. Additionally, 22.2% had somatic conditions, 24.5% reported a personal psychiatric illness, 28.2% had a family psychiatric history, 39.3% had seen a psychiatrist at least once, and 15% were using psychotropic medications. The average resilience score was 68.26 ± 14.09, with 26.3% of participants displaying low resilience. The mean self-esteem score was 29.38 ± 5.64, with 55.8% of participants exhibiting low self-esteem. No significant correlation was found between resilience and substance use. However, resilience was significantly associated with personal psychiatric illness (p < 10⁻³), family psychiatric history (p < 10⁻³), psychotropic medication use (p < 10⁻³), and psychiatrist visits (p < 10⁻³). In contrast, there was no correlation between resilience and somatic conditions. A strong association was found between resilience and self-esteem: only 6.8% of participants with normal to high self-esteem exhibited low resilience, compared to 41.8% of those with low self-esteem showing low resilience.

**Conclusions:**

This study demonstrates a significant relationship between self-esteem and resilience among Tunisian women, indicating that higher self-esteem is linked to greater resilience. These findings suggest that interventions aimed at enhancing self-esteem could improve resilience, particularly for women facing psychological difficulties. Future research could explore targeted strategies to boost self-esteem as a pathway to strengthen resilience and overall mental well-being in this population.

**Disclosure of Interest:**

None Declared

